# Characteristics of traumatic brain injury-related healthcare visits across social determinants of health: A population-based birth cohort study

**DOI:** 10.1371/journal.pone.0323902

**Published:** 2025-06-12

**Authors:** Vincy Chan, Clarissa Serafine Wirianto, Robert Balogh, Juliet Haarbauer-Krupa, Michael David Escobar

**Affiliations:** 1 KITE Research Institute, Toronto Rehabilitation Institute, University Health Network, Toronto, Ontario, Canada; 2 Faculty of Health Sciences, Ontario Tech University, Oshawa, Ontario, Canada; 3 Institute of Health Policy, Management and Evaluation, University of Toronto, Toronto, Ontario, Canada; 4 Rehabilitation Sciences Institute, Temerty Faculty of Medicine, University of Toronto, Toronto, Ontario, Canada; 5 Department of Physical Therapy, Temerty Faculty of Medicine, University of Toronto, Toronto, Ontario, Canada; 6 Division of Injury Prevention, National Center for Injury Prevention and Control, Centers for Disease Control and Prevention, Atlanta, Georgia, United States of America; 7 Dalla Lana School of Public Health, University of Toronto, Toronto, Ontario, Canada; Nationwide Children's Hospital, UNITED STATES OF AMERICA

## Abstract

**Background:**

Traumatic brain injury is a major cause of death and disability worldwide, with almost half of new cases occurring in children, adolescents, and young adults. However, data on injury characteristics stratified by social determinants of health are scarce. This study explores severity, intent, and mechanism of traumatic brain injury sustained during childhood, adolescence, and young adulthood by social determinants of health.

**Methods:**

This study utilizes a population-based birth cohort of births in publicly funded hospitals in Ontario, Canada, between April 1, 1992 and March 31, 2020 (n = 3,648,760). Individuals experiencing a traumatic brain injury requiring medical attention to the emergency department or acute care between April 1, 2002 and November 20, 2020 (n = 94,514) were identified using International Classification of Diseases Version 10 diagnosis codes. Social determinants of health variables included age, sex, rurality of residence, neighbourhood income quintile, and the following Ontario Marginalization Index variables: households and dwellings, material resources, and racialized and newcomer populations. The primary outcome was percentage of injuries falling under each mechanism, intent, and severity of injury category, stratified by social determinants of health variables.

**Results:**

Approximately 50% of injuries were mild and 96.2% of injuries were unintentional. Injury severity and intent of injury significantly varied by social determinants of health; for example, the proportion of traumatic brain injury-related healthcare visits for moderate/severe and intentional injuries was highest in areas with the lowest income quintile (13.3% and 6.1%, respectively), lowest households and dwellings stability (12.2% and 5.7%, respectively), lowest material resources (12.8% and 6.0% respectively), and highest racialized and newcomer populations (13.5% and 4.5% respectively). The percentage of traumatic brain injury-related healthcare visits for a sports-related injury significantly varied by social determinants of health; for example, the proportion of traumatic brain injury-related healthcare visits for sports-related injuries was highest among males (45.5%), those living rural areas (44.0%), and those living in areas with the highest income (47.2%), highest households and dwellings stability (44.0%), highest material resources (45.8%), and lowest racialized and newcomer populations (43.4%).

**Conclusions:**

Characteristics of traumatic brain injury-related healthcare visits vary based on social determinants of health. Targeted prevention of traumatic brain injury beyond the sports settings, including fall prevention among young children, are encouraged, and guidelines to identify and address traumatic brain injury outside of the sports setting must be developed to support early intervention of traumatic brain injury across social determinants of health.

## Introduction

Traumatic brain injury (TBI) is a leading cause of death and disability worldwide (1). In 2016, there were over 27 million new cases of TBI globally, with approximately 47% of new cases occurring in those <30 years of age [1]. A population-based birth cohort of individuals born in Ontario, Canada found that 36% of individuals in this cohort sustained a TBI that required medical attention by the age of 25 years, suggesting a significant proportion of the population who will transition or have transitioned from the pediatric to the adult health system with a history of TBI [2]. These statistics suggest that research specifically on TBI in the pediatric population is critical because TBIs sustained during childhood, adolescence, and young adulthood can compromise both skills that are developing at the time of injury and skills that are yet to develop, with challenges only becoming apparent once individuals fail to meet developmental milestones [3,4]. Even mild TBI are associated with lower quality of life, poorer academic performance, reduced participation in school, and increased healthcare utilization [5–8]. TBI is also a chronic disease with lifelong consequences, including secondary health challenges such as seizures, headaches, and endocrine dysfunction [9–11]; physical and cognitive impairments such as motor dysfunction and poor memory, attention, and executive functioning [12–14]; and social, behavioural, and emotional difficulties such as mood and anxiety disorders and deficits in communication and social language [15–17].

Despite these challenges associated with TBI, significant research and knowledge gaps exist. In particular, there is limited population-based data on the characteristics of TBI-related healthcare visits among children, youth, and young adults. For example, while data on the age and sex distribution of healthcare visits are commonly reported [1], population-based data on how TBI-related healthcare visits may differ across geographic locations (e.g., rural vs. urban, neighbourhoods characteristics such as high racialized and newcomer populations) are limited. These data are important to inform the development of targeted prevention strategies that is sensitive to the differing experiences of TBI at the community level.

This study addressed the above gaps in knowledge using data from a population-based birth cohort of individuals who sustained a TBI-related healthcare visit between 2002 and 2020 in Ontario, Canada. Specifically, individuals who sustained a TBI that required medical attention in the emergency department (ED) or hospital were identified, and information on injury characteristics (i.e., severity, intent, and mechanism) were extracted. These data were compared across age at time of injury, sex, and select social determinants of health (SDoH) variables derived from geographic location of residence (i.e., rurality of residence, neighbourhood income quintile, and neighbourhood housing and dwellings, material resources, and racialized and newcomer populations characteristics) to identify similarities and differences in injury characteristics. Findings from this study provide the foundation for the co-creation of targeted prevention of TBI at the community-level.

## Methods

Research ethics approval was obtained from the University Health Network. Informed consent was not obtained because only de-identified patient data was accessed for this study on July 30, 2021.

### Data source & sample

The population-based birth cohort was created by linking health administrative databases held at ICES. Specifically, all records of individuals born in publicly funded hospitals in Ontario, Canada, from April 1, 1992 and March 31, 2020 were identified in the MOMBABY database (N = 3,918,768). Records with stillbirths (n = 53,293), data quality issues (e.g., missing or invalid patient ID, birth date, death date, sex, n = 208,706), and ineligible for the publicly funded Ontario Health Insurance Plan (OHIP, n = 8,009) were excluded from the study. The final sample size was 3,648,760 individuals. All individuals in the birth cohort were followed up to the end of the study period (November 20, 2020), migration out of Ontario, or death. Ontario is home to 39% of Canadians and residents of Ontario have access to a universal healthcare system that covers all medically necessary services [18].

Individuals who sustained a TBI requiring medical attention (hereafter referred to as “TBI-related healthcare visit”, N = 854,412) were identified from the birth cohort using International Classification of Diseases and Related Health Problems version 10 codes (ICD-10) [19–23]. Because data on injury characteristics are only available from April 1, 2002 and onwards in the National Ambulatory Care Reporting System (NACRS), this study focused on individuals with a TBI-related healthcare visit to the ED or hospital between April 1, 2002 and the end of study period, representing 13.7% of individuals who sustained a TBI requiring medical attention. The Registered Persons Database was used to extract SDoH variables described below to explore similarities and differences across age, sex, and geographic locations. S1 Table in S1 File lists all available databases. S2 Table in S1 File lists all ICD-10 codes used to identify TBI.

### Variables

The following injury characteristics were identified: (a) injury severity, determined using the Abbreviated Injury Severity (AIS) in the Discharge Abstract Database (DAD), and using both the AIS and Glasgow Coma Scale in the NACRS [24,25]; (b) intent of injury, determined using the United States Centers for Disease Control and Prevention (CDC) Cause of Injury Matrix [26]; and (c) mechanism of injury, determined using the CDC Cause of Injury Matrix [26,27]. Sports-related injuries were also identified using the Association of Public Health Epidemiologists in Ontario categories [28].

The following SDoH variables were identified: (a) age at the time of the first TBI-related healthcare visit (hereafter referred to as the ‘index’ visit); (b) sex; (c) rurality of residence (rural vs. urban); (d) neighbourhood income quintile, and (e) neighbourhood housing and dwellings characteristic (measuring family or housing instability), determined using the Ontario Marginalization Index (ON-MARG) [29]; (f) neighbourhood material resources characteristic (measuring poverty and (in)ability for individuals and communities to access and attain basic material needs), determined using the ON-MARG [29]; and (g) neighbourhood racialized and newcomer populations characteristic (measuring recent immigrants and people belonging to a ‘visible minority’ group), determined using the ON-MARG [29]. S3 Table in S1 File lists all variables used in the study.

### Data analysis

The index TBI-related healthcare visit was the first TBI-related healthcare visit identified across the NACRS, DAD, or Ontario Mental Health Reporting System (OMHRS). Descriptive statistics of frequency distributions in the form of counts and percentages were tabulated for severity of injury, intent of injury, and mechanism of injury for the index TBI-related healthcare visit across SDoH. Bonferroni-corrected chi-squared tests with significance at p < 0.0001 were applied to test for associations between SDoH variables and injury characteristics. All diagnosis fields were considered when categorizing mechanism of injury, and thus, TBI-related healthcare visits may have more than one mechanism of injury. Records with missing SDoH were removed through pairwise deletion (<2% of individuals).

## Results

Over 90% of index TBI-related healthcare visits to the ED or acute care occurred before the age of 20 years. The majority (56.4%) of visits were among males and those living in an urban neighbourhood (81.6%) at the time of TBI. Sixteen percent of visits were among low-income neighbourhoods, 14.5% were among those living in neighbourhoods with the lowest housing and dwellings stability, 17.3% were among those living in neighbourhoods with the lowest material resources, and 15.5% were among those living in neighbourhoods with the highest racialized and newcomer population neighbourhood. Table 1 presents the sociodemographic distribution of the sample.

**Table 1:** Characteristics of individuals with a TBI-related healthcare visit in the emergency department or hospital in Ontario, Canada, April 1, 2002 to November 20, 2020 (N = 94,514)^a^.

	Total Population			Sex				
				Female		Males		p-value
	N	%	p-value	N	%	N	%	
**Overall**	94514	100.0		41233	100.0	53281	100.0	
**Sex**								
Males	53281	56.4	<0.0001	NA				
Females	41233	43.6						
**Age at Incident TBI**								
0 - 4	14402	15.2	<0.0001	6288	15.2	8114	15.2	<0.0001
5 - 9	16910	17.9		6116	14.8	10794	20.3	
10 - 14	29477	31.2		11438	27.7	18039	33.9	
15 - 19	24842	26.3		12506	30.3	12336	23.2	
20 - 24	7780	8.2		4269	10.4	3511	6.6	
25 - 28	1103	1.2		616	1.6	487	0.8	
**Rurality**								
Rural	17381	18.4	<0.0001	7526	18.3	9855	18.5	0.376
Urban	76898	81.6		33583	81.7	43315	81.5	
**Income Quintile**								
1 - Low	15473	16.5	<0.0001	6862	16.7	8611	16.2	0.050
2	16792	17.9		7403	18.0	9389	17.7	
3	18716	19.9		8188	20.0	10528	19.9	
4	20946	22.3		9004	21.9	11942	22.5	
5 - High	22113	23.5		9568	23.3	12545	23.7	
**Housing and Dwellings** ^b^								
1 - Least Marginalized	21427	23.0	<0.0001	9193	22.6	12234	23.3	<0.0001
2	20898	22.5		9086	22.4	11812	22.5	
3	19312	20.7		8261	20.3	11051	21.1	
4	17917	19.3		8008	19.7	9909	18.9	
5 - Most Marginalized	13520	14.5		6057	14.9	7463	14.2	
**Material Resources** ^b^								
1 - Least Marginalized	21949	23.6	<0.0001	9514	23.4	12435	23.7	0.670
2	21050	22.6		9137	22.5	11913	22.7	
3	18213	19.6		7959	19.6	10254	19.5	
4	15804	17.0		6931	17.1	8873	16.9	
5 - Most Marginalized	16058	17.3		7064	17.4	8994	17.1	
**Racialized and Newcomer Populations** ^b^								
1 - Least Marginalized	20514	22.0	<0.0001	9040	22.3	11474	21.9	0.004
2	20949	22.5		9123	22.5	11826	22.5	
3	19600	21.1		8693	21.4	10907	20.8	
4	17577	18.9		7632	18.8	9945	19.0	
5 - Most Marginalized	14434	15.5		6117	15.1	8317	15.9	

^a^Individuals with missing SDoH were excluded (<2% of individuals).

^b^Based on the ON-MARG.

### Injury Severity and Intent of Injury

**Fig 1:**
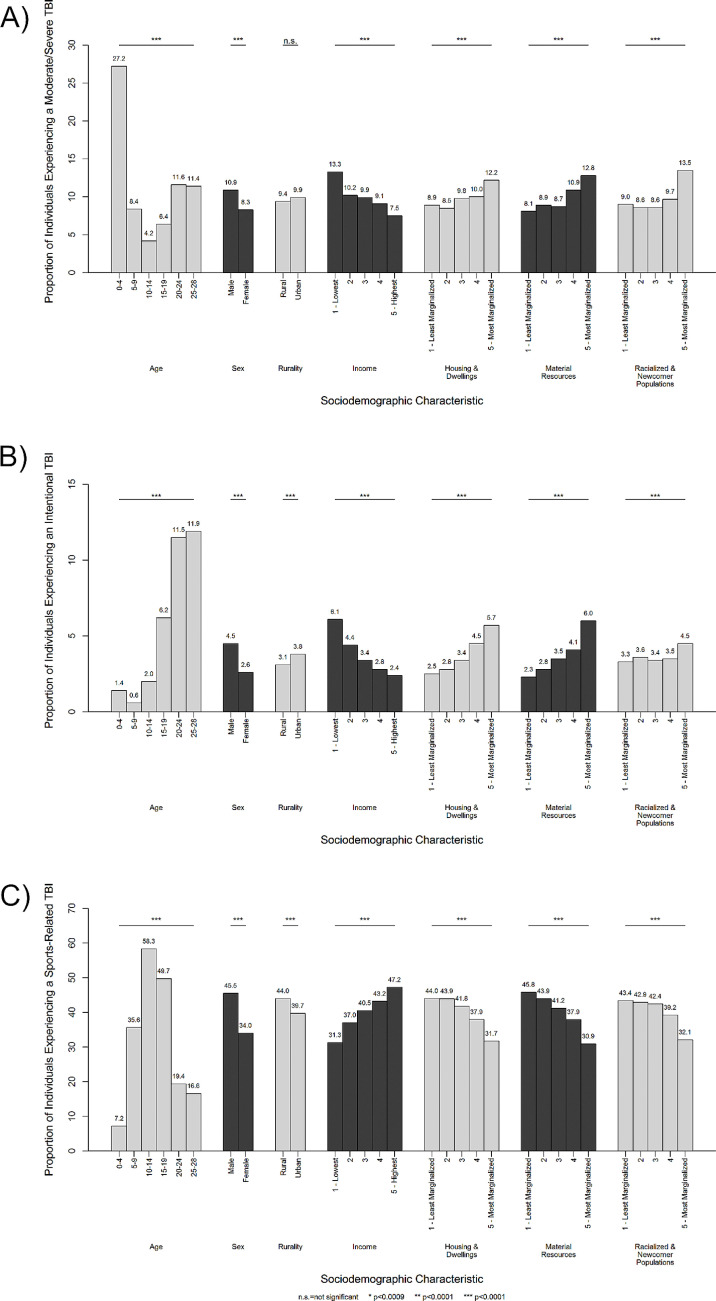
Proportion of TBI-Related Healthcare Visits by a) Moderate or severe (N = 5,110), b) intentional (N = 3,469), and c) sports-related (N** = 38,258) injuries and Social Determinants of Health Variables in Ontario, Canada, April 1, 2002 to November 20, 2020**^a,b,c^. ^a^ The proportion of moderate to severe and intentional injuries are based on known severity and intents of injury. ^b^ Households and dwellings, material resources, and racialized and newcomer populations are based on the ON-MARG. ^c^ Bonferroni-corrected chi-square p-values: n.s. = not significant, * p < 0.0009, ** p < 0.0001, *** p < 0.00001.

Overall, 49.7% of incident TBI-related healthcare visits to the ED or acute care were mild, 5.4% moderate or severe, and 44.9% were of unspecified injury severity. Among known injury severities and individuals 0–4 years of age, 27.2% of the TBI-related health care visits were of moderate or severe injury (Fig 1a). Overall, 96.2% were unintentional injuries. However, 6.2% of TBI-related healthcare visits among individuals 15–19 years old, 11.5% among 20–24 years old, and 11.9% among 25–28 years old were intentional injuries (Fig 1b). By sex, 4.5% of TBI-related healthcare visits among males were intentional injuries compared to 2.6% among females. Injury severity and intent of injury varied by SDoH; for example, the proportion of TBI-related healthcare visits that were of moderate/severe injury severity or intentional were highest among those living in neighbourhoods characterized by low income, low households and dwellings stability, low material resources, and high racialized and newcomer population. Age, sex, income quintile, housing and dwellings, material resources, and racialized and newcomer populations were significantly associated with severity of TBI (p < 0.0001). All SDoH were significantly associated with intent of injury (p < 0.0001).

### Mechanism of injury of TBI-related healthcare visits to the ED or acute care

Overall, being struck by/against an object was the most common cause of TBI-related healthcare visits (44.2%), followed by falls (40.6%), motor vehicle collision (5.8%), and other (9.4%); this distribution differed by SDoH (Table 2). S4 and S5 Tables in S1 File present these data for males and females, respectively. Overall, 40.5% of incident TBI-related healthcare visits were due to sports-related injuries. Among individuals aged 10–14 years and 15–19 years, 58.3% and 49.7% of visits, respectively, were sports-related injuries (Fig 1c). Among males, 45.5% of visits were sports-related injuries, compared to 34.0% among females. As neighbourhood income quintile increased, the proportion of TBI-related healthcare visits for sports-related injuries increased from 31.3% to 47.2%. All SDoH were significantly associated with mechanism of injury (p < 0.0001).

**Table 2:** Mechanisms of traumatic brain injury by social determinants of health variables in Ontario, Canada, April 1, 2002 to November 20, 2020 (N = 94,442)^a^^,^^b^.

	N^c^^,^^d^	% Falls	% Struck By/Against	% MVC	% Other	p-value
**Overall**	95630	40.6	44.2	5.8	9.4	
**Age at Incident TBI**						
0 - 4	14551	77.5	13.1	2.4	7.0	<0.0001
5 - 9	17154	52.4	36.0	3.0	8.6	
10 - 14	29844	33.8	54.7	2.6	8.9	
15 - 19	25113	24.2	56.0	9.1	10.7	
20 - 24	7850	26.9	41.8	18.6	12.7	
25 - 28	1118	26.7	42.5	19.8	11.0	
**Sex**						
Males	53937	39.0	46.6	4.8	9.6	<0.0001
Females	41693	42.6	41.0	7.2	9.2	
**Rurality**						
Rural	17615	38.4	44.7	5.8	11.1	<0.0001
Urban	77777	41.1	44.0	5.9	9.0	
**Income Quintile**						
1 - Low	15676	42.5	40.4	7.0	10.1	<0.0001
2	16992	40.6	43.4	6.4	9.6	
3	18951	40.6	43.9	6.0	9.5	
4	21185	40.3	45.1	5.5	9.1	
5 - High	22345	39.5	46.7	4.7	9.1	
**Housing and Dwellings** ^e^						
1 - Least Marginalized	21645	40.0	45.3	5.5	9.2	<0.0001
2	21156	38.7	45.9	5.7	9.7	
3	19546	40.3	44.4	5.7	9.6	
4	18149	41.4	43.4	6.0	9.2	
5 - Most Marginalized	13675	44.0	40.7	6.4	8.9	
**Material Resources** ^e^						
1 - Least Marginalized	22191	40.8	45.9	4.8	8.5	<0.0001
2	21277	39.8	45.4	5.4	9.4	
3	18432	40.0	44.4	6.1	9.5	
4	16007	40.3	44.0	6.2	9.5	
5 - Most Marginalized	16264	42.5	40.4	7.1	10.0	
**Racialized and Newcomer Populations** ^e^						
1 - Least Marginalized	20805	37.8	45.4	6.2	10.6	<0.0001
2	21184	38.6	45.7	5.9	9.8	
3	19829	41.3	44.7	5.1	8.9	
4	17770	42.3	43.8	5.3	8.6	
5 - Most Marginalized	14583	44.6	40.2	6.7	8.5	

**MVC:** Motor vehicle collision; **TBI:** Traumatic brain injury

^a^Mechanism of injury data were unknown for 0.1% (n = 72) of patients; this table reports only known mechanisms of injury.

^b^Data were identified from the Discharge Abstract Database, National Ambulatory Care Reporting System, and the Ontario Mental Health Reporting System databases.

^c^TBI-related healthcare visits may be due to more than one mechanism of injury; as such, total Ns do not add up to the N of total individuals.

^d^Individuals with missing SDoH were excluded (<2% of individuals).

^e^Based on the ON-MARG.

## Discussion

This population-based birth cohort study explored the injury characteristics for individuals who experienced a TBI-related healthcare visit to the ED or hospital. Findings from this study identified opportunities for targeted prevention of TBI and early intervention for TBI care across SDoH. In particular, current prevention and education measures are often focused on sport-related injuries [30] and may not be applicable to the general population. In this population-based birth cohort, only 1 in 3 TBI-related healthcare visits among females were sports-related and, consistent with recent research on mechanisms of concussion among children [31], a minority of TBI-related healthcare visits among children under the age of 10 years were sports-reported. Furthermore, a minority of TBI-related healthcare visits among individuals living in areas with the lowest income, lowest households and dwellings stability, lowest material resources, and highest racialized and newcomer population were sports-related. Conversely, the proportion of TBI-related healthcare visits among individuals in these neighbourhoods that were of moderate/severe and/or intentional injury was higher than that of individuals living in other neighbourhoods. Complementing recommendations from the Lancet Neurology Commission on TBI [32], in-depth investigation into the context of TBI among young females outside of the sports-context, particularly outside of team sports, is encouraged to inform targeted prevention of TBI among these individuals. This study also further highlights the importance of targeted falls prevention in young children [33], as the majority of TBI-related healthcare visits among children 0–4 years of age were fall-related.

In addition, given the importance of early intervention after sustaining a TBI [34], appropriate healthcare and supports within schools, communities, and the workplace must be available to address adverse outcomes associated with TBI. To date, most guidelines on TBI focus on those who sustain a TBI in the sports or school settings [35] and there is limited consideration for underserved populations in rehabilitation or clinical practice guidelines for TBI [36–38]. This study provided evidence that the proportion of TBI-related healthcare visits that were of moderate or severe or intentional injury were higher among individuals living in areas with the lowest income, lowest households and dwellings stability, lowest material resources, and highest racialized and newcomer population marginalized neighbourhoods. However, the proportion of TBI-related ED and acute care visits that were due to sports-related injuries were higher among individuals living in areas with the highest income, with the highest households and dwellings stability, highest material resources, and lowest racialized and newcomer population. As such, identification and management of TBI for underserved populations and individuals who sustain a TBI outside of the sports setting is important and encouraged to support early intervention of TBI for all individuals across diverse SDoH.

### Strengths and limitations

We acknowledge the following limitations. Only patients who sought and/or received medical care in a publicly funded hospital for a TBI in the ED or acute care were included in this study. As such, individuals with a TBI who did not seek medical care or were not coded with a TBI diagnosis code were not captured in this study. We further acknowledge limitations associated with the use of health administrative data for research. For example, because health administrative data were not collected for the purpose of research, data of interest in TBI research are not available or are limited. Specifically, injury severity data are only available for 55% of the sample, as the remaining visits were coded as ‘unspecified injury to the head’. As a result, findings from this study may represent more severe TBIs that received medical attention in the ED or acute care setting and may not be generalizable to all TBI, particular those who are only seen by primary care physicians. Similarly, we acknowledge that data from primary care physician visits are important to include, as research has found that a significant proportion of TBIs are seen in primary care settings [2]. Unfortunately, the health administrative database that captures primary care visits in Ontario, Canada, only allow for the coding of the medical reason of visit, and not details of the visit. Efforts to asses the characteristics of TBIs seen by primary care physicians are encouraged to inform opportunities to support targeted prevention and early intervention of TBI.

Despite these limitations, our study benefits from several strengths. Our study is based on a population-based birth cohort and all individuals in this sample receive medically necessary health services. The collection of these data is also mandatory in the province of Ontario in Canada. As such, all TBI-related healthcare visits to the ED or acute care setting are captured, and all data on their injury characteristics, where collected, are reported. The sample are individuals born from 1992, representing TBI-related healthcare visits in more recent decades compared to existing injury characteristics obtained from published birth cohort studies, such as the 1966 North Finland Birth Cohort [39], the 1987 Finnish Birth Cohort [40], and the 1973–1985 Swedish Birth Cohort [41]. Select SDoH data available in this birth cohort were also integrated into the analysis, providing foundational data to explore the relationship between injury characteristics and SDoH.

## Conclusion

This population-based birth cohort study addressed existing gaps in knowledge and research on TBI to provide foundational data to inform the co-creation of targeted prevention of TBI at the community-level. Findings showed a minority of females, children younger than age 10 years, and individuals living in areas with the lowest income, lowest households and dwellings stability, lowest material resources, and highest racialized and newcomer population sustained a sports-related TBI. Targeted prevention of TBI in addition to the sports settings, including falls prevention among young children, are encouraged, and guidelines to identify and address TBI outside of the sports setting must be developed to support early intervention of TBI across SDoH.

## Supporting information

S1 File S1 Table. **Data sources for the population-based birth cohort. S2 Table. International Classification of Diseases and Related Health Problems (ICD) Version 10 Codes for Traumatic Brain Injury. S3 Table. Definition of Variables Used in the Study. S4 Table. Mechanism of Traumatic Brain Injury Among Males by Social Determinants of Health Variables in Ontario, Canada, April 1, 2002 to November 20, 2020 (N = 53,236)**^a,b^. **S5 Table.** **Mechanism of Traumatic Brain Injury Among Females** **by Social Determinants of Health Variables in Ontario, Canada, April 1, 2002 to November 20, 2020 (N=41,206)** ^a,b^**. S6 Table. Severity of Injury by Social Determinants of Health Variables in Ontario, Canada, April 1, 2002 to November 20, 2020 (N = 52,049)**^a,b,c^. **S7 Table. Intent of Injury by Social Determinants of Health Variables in Ontario, Canada, April 1, 2002 to November 20, 2020 (N = 94,588)**^a,b,c^. **S8 Table. Sport-Related Injuries by Social Determinants of Health Variables in Ontario, Canada, April 1, 2002 to November 20, 2020 (N = 94,514)**^a,b^**.**
(DOCX)
