# Supplementary material for: Characteristics of traumatic brain injury-related healthcare visits across social determinants of health: A population-based birth cohort study
Source: PLoS One. 2025 Jun 12;20(6):e0323902. doi: 10.1371/journal.pone.0323902 (PMC12161583; doi:10.1371/journal.pone.0323902)
Supplement: S1 File — Data sources for the population-based birth cohort. S2 Table. International Classification of Diseases and Related Health Problems (ICD) Version 10 Codes for Traumatic Brain Injury. S3 Table. Definition of Variables Used in the Study. S4 Table. Mechanism of Traumatic Brain Injury Among Males by Social Determinants of Health Variables in Ontario, Canada, April 1, 2002 to November 20, 2020 (N = 53,236)a,b. S5 Table. Mechanism of Traumatic Brain Injury Among Females by Social Determinants of Health Variables in Ontario, Canada, April 1, 2002 to November 20, 2020 (N=41,206) a,b. S6 Table. Severity of Injury by Social Determinants of Health Variables in Ontario, Canada, April 1, 2002 to November 20, 2020 (N = 52,049)a,b,c. S7 Table. Intent of Injury by Social Determinants of Health Variables in Ontario, Canada, April 1, 2002 to November 20, 2020 (N = 94,588)a,b,c. S8 Table. Sport-Related Injuries by Social Determinants of Health Variables in Ontario, Canada, April 1, 2002 to November 20, 2020 (N = 94,514)a,b. (DOCX) [file pone.0323902.s001.docx]

**Supplementary Information**

**S1 Table. Data sources for the population-based birth cohort.**

| **Database & Description** | |
| --- | --- |
| **Population & Demographics** | **MOMBABY:** Derived from ICES (formerly Institute for Clinical Evaluative Sciences) by linking all delivering mothers’ hospitalization records to newborns. A unique de-identified patient ID is provided for the newborns. |
|  | **Registered Persons Database (RPDB):** Contains demographic data (e.g., age, sex, location of residence, date of birth and death, migration) for all residents of Ontario. |
|  | **Ontario Marginalization Index (ON-MARG):** A geographically based index derived by ICES by applying an algorithm detailed in the Canadian Marginalization Index to quantify the degree of marginalization across four major dimensions (housing and dwellings, material resources, age and labour force, and racialized and newcomer populations) in Ontario by geographic location of residence. |
| **Health Services Utilization** | **Ontario Health Insurance Plan (OHIP):** Contains data on the services provided, date of service, and primary diagnosis for all inpatient and outpatient services provided by primary physicians. |
|  | **National Ambulatory Care Reporting System (NACRS):** Contains administrative, clinical (e.g., primary and up to nine secondary diagnoses), and demographic data for all visits to publicly funded hospital- and community-based ambulatory care centres – emergency departments, hemodialysis units, and cancer care clinics – in Ontario. |
|  | **Discharge Abstract Database (DAD):** Contains administrative, clinical (e.g., most responsible diagnosis and up to 24 secondary diagnoses and procedures/interventions), and demographic information for all admissions to publicly funded acute care hospitals and day surgery institutions in Ontario. |
|  | **Ontario Mental Health Reporting System (OMHRS):** Contains administrative, clinical (e.g., diagnoses), and demographic data on all admissions to adult designated inpatient mental health beds in general hospitals, provincial psychiatric facilities, and specialty psychiatric facilities. |

**S2 Table. International Classification of Diseases and Related Health Problems (ICD) Version 10 Codes for Traumatic Brain Injury.**

| **Diagnosis Codes** | |
| --- | --- |
| **ICD** | S02.0: Fracture of vault of skull  S02.1: Fracture of base of skull  S02.3: Fracture of orbital floor  S02.7: Multiple fractures involving skull and facial bones  S02.8: Fracture of other skull and facial bones  S02.9: Fracture of skull and facial bones, part unspecified  S04.0: Injury of optic nerve and pathways  S06: Intracranial injury  S07.1: Crushing injury of skull  T02.0: Fracture involving head with neck  T06.0: Injuries of brain and cranial nerves with injuries of nerves and spinal cord at neck level |

**S3 Table. Definition of Variables Used in the Study**

| **Variable Name** | **Definition** |
| --- | --- |
| Age at TBI | Determined by the age of the first TBI-related healthcare visit |
| Sex | Determined using the RPDB |
| Rurality of residence | Determined by geographic location of residence |
| Neighbourhood income | Determined by the location of the patient’s residence within dissemination areas ranked according to income |
| Neighbourhood housing and dwellings characteristic | Determined by linking the patients’ postal code to the ON-MARG database [29], measuring family or housing instability |
| Neighbourhood material resources characteristic | Determined by linking the patients’ postal code to the ON-MARG database [29], measuring (in)ability to access and attain basic material needs |
| Neighbourhood racialized and newcomer populations characteristic | Determined by linking the patients’ postal code to the ON-MARG database [29], measuring recent immigrants and people belonging to a ‘visible minority’ group |
| Injury severity | Determined using the AIS scale [24] and, for patients identified in the NACRS database, the AIS and the Glasgow Coma Scale [25] |
| Mechanism of injury | Determined using the CDC External Cause of Injury Matrices (e.g., falls, struck by/against an object [i.e., injury resulting from being hit or crushed, or injury caused by hitting against, a human, animal, or inanimate object or force other than a vehicle or machinery [42]], motor vehicle collision, others) [26,27] |
| Intent of injury | Determined using CDC External Cause of Injury Matrices (unintentional vs. intentional – self-harm, assaults) [26] |
| Context of injury | Determined using the Association of Public Health Epidemiologists in Ontario categories of sports and recreational injuries [28] |

**AIS:** Abbreviated Injury Severity; **CDC:** Centers for Disease Control and Prevention; **DAD:** Discharge Abstract Database; **ED:** Emergency department; **NACRS:** National Ambulatory Care Reporting System; **OMHRS:** Ontario Mental Health Reporting System; **ON-MARG:** Ontario marginalization index; **RPDB:** Registered Persons Database; **TBI:** Traumatic brain injury

**S4 Table. Mechanism of Traumatic Brain Injury Among Males** **by Social Determinants of Health Variables in Ontario, Canada, April 1, 2002 to November 20, 2020 (N=53,236)**^a,b^

|  | N^c,d^ | % Falls | % Struck By/Against | % MVC | % Other | p-value |
| --- | --- | --- | --- | --- | --- | --- |
| **Overall** | 53937 | 39.0 | 46.6 | 4.8 | 9.6 |  |
| **Age at Incident TBI** | | | | | | |
| 0 - 4 | 8191 | 75.9 | 14.0 | 2.5 | 7.6 | <0.0001 |
| 5 - 9 | 10960 | 50.3 | 38.4 | 2.8 | 8.5 |  |
| 10 - 14 | 18262 | 32.4 | 56.7 | 2.1 | 8.8 |  |
| 15 - 19 | 12473 | 20.1 | 60.8 | 7.9 | 11.2 |  |
| 20 - 24 | 3557 | 22.4 | 45.4 | 17.2 | 15.0 |  |
| 25 - 28 | 494 | 22.9 | 44.5 | 19.4 | 13.2 |  |
| **Rurality** | | | | | | |
| Rural | 9991 | 36.5 | 47.3 | 4.9 | 11.3 | <0.0001 |
| Urban | 43835 | 39.6 | 46.4 | 4.8 | 9.2 |  |
| **Income Quintile** | | | | | | |
| 1 - Low | 8726 | 41.2 | 42.1 | 5.8 | 10.9 | <0.0001 |
| 2 | 9503 | 39.3 | 45.5 | 5.3 | 9.9 |  |
| 3 | 10659 | 39.1 | 46.3 | 5.0 | 9.6 |  |
| 4 | 12088 | 38.7 | 47.7 | 4.6 | 9.0 |  |
| 5 - High | 12691 | 37.7 | 49.7 | 3.8 | 8.8 |  |
| **Housing and Dwellings** ^e^ | | | | | | |
| 1 - Least Marginalized | 12367 | 38.2 | 48.5 | 4.5 | 8.8 | <0.0001 |
| 2 | 11962 | 36.9 | 48.4 | 4.9 | 9.8 |  |
| 3 | 11199 | 39.4 | 46.5 | 4.6 | 9.5 |  |
| 4 | 10034 | 39.8 | 45.6 | 5.0 | 9.6 |  |
| 5 - Most Marginalized | 7554 | 42.5 | 42.6 | 5.2 | 9.7 |  |
| **Material Resources** ^e^ | | | | | | |
| 1 - Least Marginalized | 12578 | 39.0 | 49.0 | 3.9 | 8.1 | <0.0001 |
| 2 | 12047 | 38.0 | 48.1 | 4.5 | 9.4 |  |
| 3 | 10399 | 38.6 | 46.7 | 5.2 | 9.5 |  |
| 4 | 8984 | 38.6 | 46.5 | 5.2 | 9.7 |  |
| 5 - Most Marginalized | 9108 | 41.5 | 41.8 | 5.7 | 11.0 |  |
| **Racialized and Newcomer Populations** ^e^ | | | | | | |
| 1 - Least Marginalized | 11645 | 35.9 | 47.8 | 5.5 | 10.8 | <0.0001 |
| 2 | 11959 | 37.1 | 48.4 | 4.8 | 9.7 |  |
| 3 | 11049 | 40.0 | 47.2 | 4.0 | 8.8 |  |
| 4 | 10057 | 40.6 | 46.6 | 4.1 | 8.7 |  |
| 5 - Most Marginalized | 8406 | 43.3 | 41.9 | 5.8 | 9.0 |  |

**MVC:** Motor vehicle collision; **TBI:** Traumatic brain injury

^a^ Mechanism of injury data were unknown for 0.1% (n=45) of patients; this table reports only known mechanisms of injury.

^b^ Data were identified from the Discharge Abstract Database, National Ambulatory Care Reporting System, and the Ontario Mental Health Reporting System databases.

^c^ TBI-related healthcare visits may be due to more than one mechanism of injury; as such, total Ns do not add up to the N of total individuals.

^d^ Individuals with missing SDoH were excluded (<2% of individuals).

^e^ Based on the ON-MARG.

**S5 Table. Mechanism of Traumatic Brain Injury Among Females** **by Social Determinants of Health Variables in Ontario, Canada, April 1, 2002 to November 20, 2020 (N=41,206)**^a,b^

|  | N^c,d^ | % Falls | % Struck By/Against | % MVC | % Other | p-value |
| --- | --- | --- | --- | --- | --- | --- |
| **Overall** | 41693 | 42.6 | 41.0 | 7.2 | 9.2 |  |
| **Age at Incident TBI** | | | | | | |
| 0 - 4 | 6360 | 79.7 | 12.1 | 2.2 | 6.0 | <0.0001 |
| 5 - 9 | 6194 | 56.2 | 31.7 | 3.4 | 8.7 |  |
| 10 - 14 | 11582 | 36.0 | 51.5 | 3.3 | 9.2 |  |
| 15 - 19 | 12640 | 28.2 | 51.2 | 10.2 | 10.4 |  |
| 20 - 24 | 4293 | 30.6 | 38.8 | 19.7 | 10.9 |  |
| 25 - 28 | 624 | 29.6 | 40.9 | 20.0 | 9.5 |  |
| **Rurality** | | | | | | |
| Rural | 7624 | 40.9 | 41.2 | 6.9 | 11.0 | <0.0001 |
| Urban | 33942 | 43.0 | 41.0 | 7.2 | 8.8 |  |
| **Income Quintile** | | | | | | |
| 1 - Low | 6950 | 44.1 | 38.2 | 8.5 | 9.2 | <0.0001 |
| 2 | 7489 | 42.4 | 40.7 | 7.9 | 9.0 |  |
| 3 | 8292 | 42.6 | 40.9 | 7.4 | 9.1 |  |
| 4 | 9097 | 42.5 | 41.7 | 6.7 | 9.1 |  |
| 5 - High | 9654 | 42.0 | 42.8 | 5.8 | 9.4 |  |
| **Housing and Dwellings** ^e^ | | | | | | |
| 1 - Least Marginalized | 9278 | 42.5 | 41.2 | 6.9 | 9.4 | <0.0001 |
| 2 | 9194 | 41.1 | 42.6 | 6.8 | 9.5 |  |
| 3 | 8347 | 41.6 | 41.5 | 7.2 | 9.7 |  |
| 4 | 8115 | 43.3 | 40.6 | 7.2 | 8.9 |  |
| 5 - Most Marginalized | 6121 | 45.8 | 38.4 | 8.0 | 7.8 |  |
| **Material Resources** ^e^ | | | | | | |
| 1 - Least Marginalized | 9613 | 43.1 | 41.9 | 6.1 | 8.9 | <0.0001 |
| 2 | 9230 | 42.2 | 41.9 | 6.6 | 9.3 |  |
| 3 | 8033 | 41.8 | 41.4 | 7.4 | 9.4 |  |
| 4 | 7023 | 42.4 | 40.8 | 7.4 | 9.4 |  |
| 5 - Most Marginalized | 7156 | 43.9 | 38.6 | 8.8 | 8.7 |  |
| **Racialized and Newcomer Populations** ^e^ | | | | | | |
| 1 - Least Marginalized | 9160 | 40.4 | 42.3 | 7.1 | 10.2 | <0.0001 |
| 2 | 9225 | 40.6 | 42.3 | 7.3 | 9.8 |  |
| 3 | 8780 | 42.9 | 41.5 | 6.7 | 8.9 |  |
| 4 | 7713 | 44.5 | 40.0 | 6.9 | 8.6 |  |
| 5 - Most Marginalized | 6177 | 46.4 | 37.9 | 8.1 | 7.6 |  |

**MVC:** Motor vehicle collision; **TBI:** Traumatic brain injury

^a^ Mechanism of injury data were unknown for 0.1% (n=27) of patients; this table reports only known mechanisms of injury.

^b^ Data were identified from the Discharge Abstract Database, National Ambulatory Care Reporting System, and the Ontario Mental Health Reporting System databases.

^c^ TBI-related healthcare visits may be due to more than one mechanism of injury; as such, total Ns do not add up to the N of total individuals.

^d^ Individuals with missing SDoH were excluded (<2% of individuals).

^e^ Based on the ON-MARG.

**S6 Table. Severity of Injury** **by Social Determinants of Health Variables in Ontario, Canada, April 1, 2002 to November 20, 2020 (N=52,049)**^a,b,c^

|  | **Mild** | | **Moderate & Severe** | | **p-value** |
| --- | --- | --- | --- | --- | --- |
|  | **N** | **%** | **N** | **%** |  |
| **Overall** | 46939 | 100 | 5110 | 100 |  |
| **Sex** | | | | | |
| Males | 27032 | 57.59 | 3315 | 64.87 | <0.0001 |
| Females | 19907 | 42.41 | 1795 | 35.13 |  |
| **Age at Incident TBI** | | | | | |
| 0 - 4 | 5923 | 12.62 | 2208 | 43.21 | <0.0001 |
| 5 - 9 | 9538 | 20.32 | 878 | 17.18 |  |
| 10 - 14 | 15775 | 33.61 | 689 | 13.48 |  |
| 15 - 19 | 11528 | 24.56 | 787 | 15.4 |  |
| 20 - 24 | 3586 | 7.64 | 472 | 9.24 |  |
| 25 - 28 | 589 | 1.25 | 76 | 1.49 |  |
| **Rurality** | | | | | |
| Rural | 8049 | 17.19 | 838 | 16.45 | 0.191 |
| Urban | 38780 | 82.81 | 4256 | 83.55 |  |
| **Income Quintile** | | | | | |
| 1 - Low | 7584 | 16.23 | 1164 | 22.95 | <0.0001 |
| 2 | 8313 | 17.79 | 948 | 18.69 |  |
| 3 | 9210 | 19.71 | 1014 | 19.99 |  |
| 4 | 10371 | 22.2 | 1036 | 20.42 |  |
| 5 - High | 11242 | 24.06 | 911 | 17.96 |  |
| **Housing and Dwellings** ^d^ | | | | | |
| 1 - Least Marginalized | 10770 | 23.27 | 1053 | 21.16 | <0.0001 |
| 2 | 10419 | 22.51 | 973 | 19.55 |  |
| 3 | 9405 | 20.32 | 1017 | 20.44 |  |
| 4 | 8875 | 19.18 | 984 | 19.77 |  |
| 5 - Most Marginalized | 6811 | 14.72 | 949 | 19.07 |  |
| **Material Resources** ^d^ | | | | | |
| 1 - Least Marginalized | 11328 | 24.48 | 1000 | 20.1 | <0.0001 |
| 2 | 10193 | 22.02 | 995 | 20 |  |
| 3 | 8965 | 19.37 | 851 | 17.1 |  |
| 4 | 7756 | 16.76 | 951 | 19.11 |  |
| 5 - Most Marginalized | 8038 | 17.37 | 1179 | 23.69 |  |
| **Racialized and Newcomer Populations** ^d^ | | | | | |
| 1 - Least Marginalized | 9612 | 20.77 | 948 | 19.05 | <0.0001 |
| 2 | 10265 | 22.18 | 964 | 19.37 |  |
| 3 | 9892 | 21.37 | 936 | 18.81 |  |
| 4 | 9148 | 19.77 | 980 | 19.69 |  |
| 5 - Most Marginalized | 7363 | 15.91 | 1148 | 23.07 |  |

^a^ Severity of injury data were unknown for 44.9% (n=42,465) of patients; this table reports only known severities of injury.

^b^ Data were identified from the Discharge Abstract Database, National Ambulatory Care Reporting System, and the Ontario Mental Health Reporting System databases.

^c^ Individuals with missing SDoH were excluded (<2% of individuals).

^d^ Based on the ON-MARG.

**S7 Table. Intent of Injury** **by Social Determinants of Health Variables in Ontario, Canada, April 1, 2002 to November 20, 2020 (N=94,588)**^a,b,c^

|  | **Intentional** | | **Unintentional & Others** | | **p-value** |
| --- | --- | --- | --- | --- | --- |
|  | **N** | **%** | **N** | **%** |  |
| **Overall** | 3469 | 100 | 91119 | 100 |  |
| **Sex** | | | | | |
| Males | 2384 | 68.72 | 50947 | 68.24 | <0.0001 |
| Females | 1085 | 31.28 | 40172 | 31.76 |  |
| **Age at Incident TBI** | | | | | |
| 0 - 4 | 200 | 5.77 | 14233 | 5.92 | <0.0001 |
| 5 - 9 | 106 | 3.06 | 16801 | 3.17 |  |
| 10 - 14 | 599 | 17.27 | 28889 | 17.11 |  |
| 15 - 19 | 1544 | 44.51 | 23330 | 44.44 |  |
| 20 - 24 | 888 | 25.6 | 6890 | 25.51 |  |
| 25 - 28 | 132 | 3.81 | 976 | 3.86 |  |
| **Rurality** | | | | | |
| Rural | 531 | 15.41 | 16864 | 15.33 | <0.0001 |
| Urban | 2914 | 84.59 | 74041 | 84.67 |  |
| **Income Quintile** | | | | | |
| 1 - Low | 949 | 27.62 | 14560 | 27.7 | <0.0001 |
| 2 | 736 | 21.42 | 16073 | 21.46 |  |
| 3 | 642 | 18.68 | 18089 | 18.46 |  |
| 4 | 581 | 16.91 | 20373 | 17.04 |  |
| 5 - High | 528 | 15.37 | 21580 | 15.34 |  |
| **Housing and Dwellings** ^d^ | | | | | |
| 1 - Least Marginalized | 525 | 15.7 | 20900 | 15.78 | <0.0001 |
| 2 | 594 | 17.76 | 20319 | 17.81 |  |
| 3 | 654 | 19.55 | 18668 | 19.41 |  |
| 4 | 802 | 23.98 | 17140 | 24.06 |  |
| 5 - Most Marginalized | 770 | 23.02 | 12773 | 22.95 |  |
| **Material Resources** ^d^ | | | | | |
| 1 - Least Marginalized | 505 | 15.1 | 21439 | 15.18 | <0.0001 |
| 2 | 581 | 17.37 | 20470 | 17.55 |  |
| 3 | 640 | 19.13 | 17591 | 19.12 |  |
| 4 | 656 | 19.61 | 15161 | 19.32 |  |
| 5 - Most Marginalized | 963 | 28.79 | 15139 | 28.84 |  |
| **Racialized and Newcomer Populations** ^d^ | | | | | |
| 1 - Least Marginalized | 678 | 20.27 | 19849 | 20.21 | <0.0001 |
| 2 | 745 | 22.27 | 20210 | 21.89 |  |
| 3 | 661 | 19.76 | 18955 | 19.95 |  |
| 4 | 616 | 18.42 | 16970 | 18.41 |  |
| 5 - Most Marginalized | 645 | 19.28 | 13816 | 19.55 |  |

^a^ Intent of injury data were unknown for 0.1% (n=72) of patients; this table reports only known intents of injury.

^b^ Data were identified from the Discharge Abstract Database, National Ambulatory Care Reporting System, and the Ontario Mental Health Reporting System databases.

^c^ Individuals with missing SDoH were excluded (<2% of individuals).

^d^ Based on the ON-MARG.

**S8 Table. Sport-Related Injuries** **by Social Determinants of Health Variables in Ontario, Canada, April 1, 2002 to November 20, 2020 (N=94,514)**^a,b^

|  | **Yes** | | **No** | | **p-value** |
| --- | --- | --- | --- | --- | --- |
|  | **N** | **%** | **N** | **%** |  |
| **Overall** | 38258 | 100 | 56256 | 100 |  |
| **Sex** | | | | | |
| Males | 24219 | 63.3 | 29062 | 51.66 | <0.0001 |
| Females | 14039 | 36.7 | 27194 | 48.34 |  |
| **Age at Incident TBI** | | | | | |
| 0 - 4 | 1032 | 2.7 | 13370 | 23.77 | <0.0001 |
| 5 - 9 | 6017 | 15.73 | 10893 | 19.36 |  |
| 10 - 14 | 17180 | 44.91 | 12297 | 21.86 |  |
| 15 - 19 | 12336 | 32.24 | 12506 | 22.23 |  |
| 20 - 24 | 1510 | 3.95 | 6270 | 11.15 |  |
| 25 - 28 | 183 | 0.48 | 920 | 1.64 |  |
| **Rurality** | | | | | |
| Rural | 7639 | 20 | 9742 | 17.37 | <0.0001 |
| Urban | 30557 | 80 | 46341 | 82.63 |  |
| **Income Quintile** | | | | | |
| 1 - Low | 4849 | 12.72 | 10624 | 18.99 | <0.0001 |
| 2 | 6210 | 16.3 | 10582 | 18.92 |  |
| 3 | 7574 | 19.87 | 11142 | 19.92 |  |
| 4 | 9041 | 23.72 | 11905 | 21.29 |  |
| 5 - High | 10435 | 27.38 | 11678 | 20.88 |  |
| **Housing and Dwellings** ^c^ | | | | | |
| 1 - Least Marginalized | 9430 | 24.99 | 11997 | 21.68 | <0.0001 |
| 2 | 9173 | 24.31 | 11725 | 21.19 |  |
| 3 | 8069 | 21.38 | 11243 | 20.32 |  |
| 4 | 6789 | 17.99 | 11128 | 20.11 |  |
| 5 - Most Marginalized | 4280 | 11.34 | 9240 | 16.7 |  |
| **Material Resources** ^c^ | | | | | |
| 1 - Least Marginalized | 10048 | 26.62 | 11901 | 21.51 | <0.0001 |
| 2 | 9238 | 24.48 | 11812 | 21.35 |  |
| 3 | 7504 | 19.88 | 10709 | 19.35 |  |
| 4 | 5994 | 15.88 | 9810 | 17.73 |  |
| 5 - Most Marginalized | 4957 | 13.13 | 11101 | 20.06 |  |
| **Racialized and Newcomer Populations** ^c^ | | | | | |
| 1 - Least Marginalized | 8910 | 23.61 | 11604 | 20.97 | <0.0001 |
| 2 | 8989 | 23.82 | 11960 | 21.61 |  |
| 3 | 8317 | 22.04 | 11283 | 20.39 |  |
| 4 | 6897 | 18.27 | 10680 | 19.3 |  |
| 5 - Most Marginalized | 4628 | 12.26 | 9806 | 17.72 |  |

^a^ Data were identified from the Discharge Abstract Database, National Ambulatory Care Reporting System, and the Ontario Mental Health Reporting System databases.

^b^ Individuals with missing SDoH were excluded (<2% of individuals).

^c^ Based on the ON-MARG.
